# Ozone Diffusion through a Hollow Fiber Membrane Contactor for Pharmaceuticals Removal and Bromate Minimization

**DOI:** 10.3390/membranes13020171

**Published:** 2023-01-31

**Authors:** Alice Schmitt, Julie Mendret, Hani Cheikho, Stephan Brosillon

**Affiliations:** IEM, University of Montpellier, CNRS, ENSCM, 34095 Montpellier, France

**Keywords:** membrane contactor, ozonation, PTFE, micropollutant, by-product, hydroxyl radical

## Abstract

Recently, ozonation has been advocated as a solution to tackle emerging contaminants. Hollow fiber membrane contactors (HFMC) have a lower residual ozone concentration than bubble reactors that could limit the formation of potential ozonation by-products, especially bromates that are regulated in drinking water. The aim of this study was to evaluate ozonation with HFMC for pharmaceutical abatement and bromate minimization compared to bubble columns in wastewater. A HFMC, composed of 65 polytetrafluoroethylene hollow fibers with a 0.45 mm/0.87 mm inner/external diameter and a 0.107 m² exchange surface, was used for the ozonation of real-treated wastewater spiked with 2 µM of *p*-chlorobenzoic acid (*p*-CBA) and 3 mg.L^−1^ of bromide. *p*-CBA was tracked to monitor the production of strongly-oxidant hydroxyl radicals from the decomposition of the molecular ozone. At 100% *p*-CBA abatement, 1600 µg.L^−1^ of bromate was formed with the HFMC, whereas 3486 µg.L^−1^ was formed with the bubble column. These results demonstrate that HFMC can produce a significant amount of hydroxyl radicals while limiting bromate formation in real-treated wastewater. The test water was also spiked with carbamazepine and sulfamethoxazole to evaluate the abatement efficiency of the process. Short contact times (approximately 2s) achieved high rates of pharmaceuticals removal without bromate formation.

## 1. Introduction 

The rising presence and accumulation of harmful micropollutants (MPs) in natural waters is a major environmental concern. MPs detected in the environment at very low concentrations (at a scale of µg/L down to ng/L) can, nevertheless, have damaging negative effects on living organisms due to their toxicity, persistency or bioaccumulation. However, the impact of MPs on human health and the ecosystem and particularly their additive effect, remains unknown [1,2]. Chronic exposure via drinking water consumption could, therefore, have disastrous consequences on health. In order to preserve water resources, wastewater treatment plants (WWTPs), which are one of the main sources of release of organic MPs into the receiving aquatic environment, need to be upgraded by adding advanced treatment processes to ensure these pollutants are eliminated [3]. There are several subcategories of MPs released by WWTPs, the biggest of which is pharmaceuticals [4] whose presence in aqueous environments is due to therapeutic drugs and personal hygiene products, but also hospital and pharmaceutical industry effluents [5,6]. Numerous studies have reported the presence in pharmaceutical MPs in WWTP effluents and also in surface waters around the world [7,8,9,10,11].

Carbamazepine (CBZ) is an antiepileptic drug that is widely used in many countries and is little or not at all removed by conventional WWTPs [8,12]. CBZ is thus widely found in secondary treatment stages of WWTP effluents [1,13,14,15], and studies have also reported CBZ in surface waters [8,11,16]. Likewise, sulfamethoxazole (SUL) is an antibiotic that is frequently found in WWTP effluents and in surface waters worldwide [7,8,9,10,11]. 

WWTPs, therefore, need to be upgraded by adding an appropriate advanced treatment stage for MPs abatement. One potential and economic option is ozonation. Under some experimental conditions, ozonation has a significant cost. However, for low specific doses inferior to 0.5 mgO_3_/mgC, its cost is suitable and less expensive than Granular Activated Carbon (GAC) [17]. In recent decades, ozonation processes have been successfully used to remove organic MPs, sometimes in combination with another process used as a polishing step [18,19,20,21,22,23,24]. Ozonation processes combine the strong oxidizing power of molecular ozone with the even stronger oxidizing power of the hydroxyl radicals produced from decomposition of ozone, enabling these processes to remove most organic compounds [25]. In addition, the reaction rate constants of the organic MPs with hydroxyl radicals in these processes are generally significantly higher than those with molecular ozone (i.e., k_O3_ = 10^5^ M^−1^.s^−1^ for the most reactive compounds, and k_OH_ is often higher than 10^9^ M^−1^.s^−1^) [26]. However, in the ozonation processes, the matrix is a factor as it scavenges hydroxyl radicals due to the natural organic matter and carbonates present in the water. A given matrix may require a higher ozone dose [27].

Conventional ozonation processes (e.g., ozonation rooms) employ ozone dispersion in the form of bubbles, which has several disadvantages, such as the generation of by-products that can sometimes prove more dangerous than the initial products [28]. For instance, ozonation of waters that contain bromides produces bromates, which are regulated in drinking water due to their carcinogenic potential [29,30]. The EU and the European US EPA set a limit cap of 10 μg.L^−1^ bromate in drinking water [31,32]. The presence of bromates is due to an excess of residual ozone, which is caused by the difficulty controlling the ozone dosage in conventional processes [30,33]. A promising solution could be to use membrane contactors, which could transfer the ozone in small quantities through masses of membrane pores, and thus simultaneously remove MPs while minimizing the formation of bromates [27,34,35]. The membrane acts as a barrier between the liquid and the gas phases and the mass transfer occurs by diffusion (and not by dispersion) due to a concentration gradient [36]. The ozone, therefore, gets uniformly transferred to the water to be treated via a bubbleless process.

To the best of the authors’ knowledge, only two studies have examined the formation of bromate during ozonation through an in/out membrane contactor. Merle et al. [34] studied the performance of an ozonation process with ozone and peroxone (i.e., O_3_/H_2_O_2_) on para-chlorobenzoic acid (p-CBA), which is a highly HO°-reactive and O_3_-resistant compound, through a polytetrafluoroethylene (PTFE) hollow fiber membrane contactor. In order to evaluate bromate formation during the ozonation process, each matrix (i.e., a groundwater, a river water and a lake water) was spiked with bromides and p-CBA. Merle et al. thus showed p-CBA abatement and bromate formation for different ozone gas concentrations, liquid residence times and H_2_O_2_ doses. p-CPA removal rates and bromate concentrations both increased with higher residence times and higher ozone gas concentrations. The highest H_2_O_2_ dose led to better MP abatement but also to higher bromate formation. Merle et al. also compared performances between the membrane contactor and the conventional peroxone process (gas bubble injection). Results between the two processes varied depending on the conditions tested. When ozone concentration in the gas phase was above 10 g.Nm^−3^, the conventional process outperformed the membrane contactor on groundwater, irrespective of the conditions. On lake water, the membrane contactor outperformed the conventional process at up to 5 g.Nm^−3^ of ozone in the gas phase. On river water, less bromates were produced with the membrane contactor for a p-CBA removal greater that 80%. Note, however, that for an ozone gas concentration up to 5 g.Nm^−3^, the bromate concentration never exceeded 10 µg.L^−1^ (i.e., the regulatory limit in EU), irrespective of the matrix and conditions studied. Stylianou et al. investigated the ozonation of four micropollutants (i.e., carbamazepine, benzotriazole, p-CBA and atrazine) with O_3_ and H_2_O_2_ via a ceramic tubular membrane contactor [35]. The matrix (i.e., river water from Aliakmonas River, Greece) was spiked with the four selected micropollutants. They showed that the addition of peroxone had a variable effect (i.e., positive or negative) on MPs abatement depending on the compound, and that the membrane contactor produced a high concentration of bromates (higher than the regulatory limit of 10 μg.L^−1^). They compared results obtained with conventional systems against two membrane contactors of different inner diameters (and exchange surfaces). Stylianou et al. concluded that membrane contactors should have the highest possible inner surface per volume and that a low ozone concentration in the gas was required in order to minimize bromate formation.

This brief look at the state of art finds that efforts are still needed to evaluate the ozonation process when using membrane contactors for applications in tertiary water treatment, and specifically for the treatment of emerging pollutants. Here, to address this gap, we evaluated the performance of an in/out PTFE hollow fiber membrane contactor on the removal of two pharmaceuticals (i.e., carbamazepine and sulfamethoxazole) in a real wastewater (La Grande-Motte, France). The in/out configuration (i.e., the gas circulation was inside the fibers and the liquid circulation inside the shell) has attracted very little work [37,38] yet if offers the benefit of less risk of membrane fouling due to the avoidance of the circulation of the treated wastewater, which could carry suspended matter, in the fibers whose diameter is smaller than 1 mm. The matrix was spiked with the targeted compounds in order to overcome the constraints associated with limits of detection (LOD) and limits of quantification (LOQ) during the analyses. The second part of the study concerns bromate formation. We compared experiments done with the membrane contactor against experiments done with a semi-batch bubble reactor in the same conditions. For this second part of the study, the matrix (i.e., WWTP effluent) was spiked with bromides (LOQ and LOD constraints) and p-CBA in order to monitor the production of strongly-oxidant hydroxyl radicals from the decomposition of molecular ozone. The membrane contactor used had previously been fully characterized in an original article [39]. 

## 2. Materials and Methods

### 2.1. Chemicals

Only high-purity (>98%) analytical-grade chemicals were used, all from Sigma-Aldrich (Saint-Quentin-Fallavier, France). 

Two pharmaceuticals (carbamazepine (CBZ) and sulfamethoxazole (SUL)) were purchased from Sigma-Aldrich. Table 1 presents their main characteristics. CBZ was stored at 4 °C whereas SUL was stored at room temperature.

### 2.2. Matrix Studied

#### 2.2.1. Real WWTP Effluent

Real-treated wastewater coming from a seaside-resort WWTP (La Grande-Motte, France) was used as a complex matrix. This WWTP has a maximum capacity of 65,000 population-equivalents and treats 5702 m^3^.d^−1^ of domestic wastewater on average, depending on the season, due to the 5-fold increase in the population during the summer. The WWTP performs nitrogen removal via to a biological nitrification/denitrification process with anaerobic zones alternating with anoxia zones. The WWTP is then equipped with an activated sludge process in an aerated basin (medium load), followed by a unique configuration at the outlet involving a membrane bioreactor (MBR) with submerged membrane units (SMU RW400) (KUBOTA, Japan) composed of flat-sheet microporous membranes made of chlorinated polyethylene. The membranes have a total surface of 16,240 m² and an average pore size of 0.2 µm (ultrafiltration). Table 2 and Table 3 report the characteristics of the MBR permeate corresponding to the WWTP effluent. In order to optimize wastewater treatment according to season (high flow-rate during summer and low load during winter), two identical channels are present upstream of the MBR (5770 m^3^ per channel), the second of which is only applied in the summer season. The sewage sludge is treated by static gravity thickening and amounts to 345.48 tons of dry matter per year. The effluent was sampled at the outlet of the MBR and stored at 4 °C after sampling in order to minimize variation in its composition.

#### 2.2.2. Solution Spiked with the Targeted MP

A volume of 30 L of the real matrix (i.e., real-treated wastewater) was spiked with 2 mg.L^−1^ of each MP studied. This solution was then agitated for at least 24 h (to ensure complete dissolution of the MPs) before being incorporated in the feed tank. The solution was then balanced with the system (i.e., the ozonation pilot) by liquid recirculation for about 12h with compressed air (whose composition was identical to that of air) circulating inside the fibers to prevent water penetrating into the membrane pores. This balance was established in order to allow potential MP adsorption in the system (depending on the n-octanol/water partition coefficient K_OW_ of the MP). The initial concentration of each MP was then measured by analyzing three samples from the feed tank (after balancing) and three samples from the tap upstream of the membrane contactor. The average value of these samples served as initial concentration of each MP for interpretation of the results.

#### 2.2.3. Bromide Solution

The real matrix was spiked with 2 µM of p-CBA and 3 mg.L^−1^ of bromide (Br^−^) as BrNa. The solution was then agitated for at least 24h to ensure complete dissolution of the chemicals.

Note that the initial concentration of bromide in the matrix before being spiked was significantly higher during the summer (up to 1.6 mg.L^−1^) than the winter (0.3 mg.L^−1^ in average). Indeed, the water used in this work came from the WWTP outlet of a big summer seaside resort. In all the experiments, we spiked the effluents with the same quantity of bromides, irrespective of the initial bromide concentration. Consequently, and due to the organization of our experiments over time, the initial concentration of bromide was lower for the experiments with the membrane contactor than with the bubble reactor. This difference was taken into account in the interpretation of the experimental results by normalizing the bromate and bromide concentrations to the initial bromide concentration. Nevertheless, a higher initial concentration of bromide promotes the production of more bromates due to the bromide concentration-dependent kinetics of bromate formation.

### 2.3. Analytical Methods

#### 2.3.1. Ozone Analysis

The indigo method [43] was used to determine dissolved ozone concentration in the liquid phase. 

#### 2.3.2. MP Analyses

The mix of MPs was quantified by liquid chromatography–tandem mass spectrometry (LC-MS/MS) using a Waters^®^ device. An XSelect^®^ HSS-T3-C18 resin column (100 mm × 21 mm) with a 2.5 µm particle size was used as stationary phase at room temperature, with eluent A (90% HPLC-grade water + 10% HPLC-grade acetonitrile (ACN) + 0.1% formic acid) and eluent B (ACN + 0.1% formic acid). The flow-rate was 0.25 mL.min^−1^ and the gradient elution profile described in Table S1 was applied. To achieve the best sensitivity, the mass spectrometry protocol was adjusted to facilitate the ionization process, and the detection conditions were capillary potential 3.5 kV, cone voltage 30 V, source temperature 120 °C, desolvation temperature 450 °C, cone gas flow 50 NL.h^−1^, desolvation gas flow 500 NL.h^−1^ and collision energy 10 V. Nebulizer gas was nitrogen and collision gas was argon. The calibration curves were plotted on the same matrix as the samples to avoid matrix effects on detection (i.e., calibration curves were done using WWTP effluent with known concentrations of MPs). Two calibration curves were plotted for each MP by analyzing standard samples before and after analyte samples in order to avoid instrumental drift. LOD and LOQ were about 3 µg.L^−1^ and 10 µg.L^−1^ , respectively, for CBZ and 0.13 µg.L^−1^ and 0.74 µg.L^−1^, respectively, for the SUL.

The abatement of each MP was then calculated from the following expression:

(1) $$Removal\;\left(\%\right)=\frac{{\left[MP\right]}_{initial}-{\left[MP\right]}_{final}}{{\left[MP\right]}_{initial}}\times 100$$

where *[MP]_initial_* is the initial concentration and *[MP]_final_* is the final concentration of the targeted *MP*.

#### 2.3.3. p-CBA Analysis 

Analyses of p-CBA were performed by HPLC-UV on a Waters Acquity UPLC system running Empower analyst software, fitted with a NucleoShell (Macherey-Nagel) column (50-mm length × 2-mm inside diameter, 2.7-µm particle size) at room temperature (T = 22 °C). The mobile phase was buffer A (HPLC-grade water + 0.1% (*v*/*v*) trifluoroacetic acid) and buffer B (HPLC-grade ACN + 0.1% (*v*/*v*) trifluoroacetic acid). Flow rate was 0.25 mL.min^−1^. A 3-min isocratic run was applied with 80% of A and 20% of B. p-CBA retention time was 1.88 min. UV detection was performed at λ = 240 nm. 

For the samples collected after ozonation, 200 µL of Na_2_SO_3_ (10 mg.L^−1^) was added to 2 mL of sample as scavenger of the dissolved ozone.

#### 2.3.4. Characterization of Real WWTP Effluent/Global Indicator for Pollution Monitoring

pH analyses were performed with a titrator pHmeter (Titroline Easy, Schott Instruments) calibrated with two buffer solutions (pH = 4.0 and 6.87). Chemical Oxygen Demand (COD) values were measured using LCK 1414 Hach kits (5–60 mgO_2_/L).

Total Organic Carbon (TOC) analyses were performed using a Shimadzu TOC-VCSN analyzer after first passing the samples through a 0.45 µm filter. UV254 absorbance and specific UV absorbance at 254 nm (SUVA_254_) were used to track a relative amount of unsaturated and/or aromatic carbon of natural organic matter (NOM) [44]. SUVA_254_ was determined from the ratio of UV254 absorbance to TOC value. UV254 absorbance was measured in a 1 cm quartz cuvette using a Shimadzu model UV-2401PC UV-vis spectrophotometer (Shimadzu, Japan). 

Concentrations of anions and cations were measured using an ICS-1000 ion chromatograph equipped with an AERS suppressor (4 mm), an IonPac AS19 column and a DS6 conductivity detector for anions and an ICS-900 equipped with a CSRS suppressor (4 mm), an IonPac CS12A column and a DS5 conductivity detector for cations (Dionex, Thermo Scientific, Waltham, MA, USA). Samples were added automatically using a sample changer (AS40). 

For the samples collected after ozonation, 200 µL of Na_2_SO_3_ (10 mg.L^−1^) was added to 2 mL of sample as scavenger of the dissolved ozone.

### 2.4. Membrane Contactor Technology

**Figure 1:**
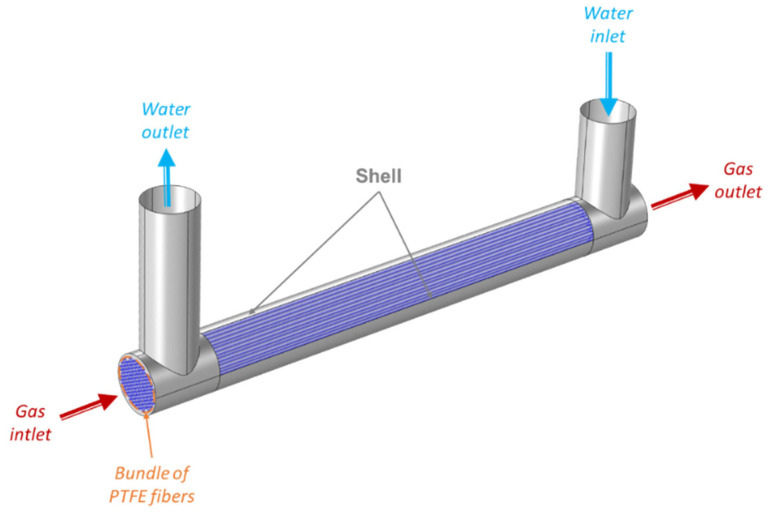
Configuration of the membrane contactor.

The PTFE hollow fibers membrane contactor used in this work was chosen to resist ozone over time and supplied by Polymem (France) [45]. Table 4 lists its key characteristics. The pilot was working via counter-current flow with gas circulating inside the fibers and liquid in the shell (Figure 1).

The membrane porosity ε (^a^) was calculated with a Sartorius CPA 225D balance as [46]:

(2) $$\varepsilon =\frac{\left(w_{\mathrm{wet}}-w_{\mathrm{dry}}\right)/D_{\mathrm{iso}}}{\frac{w_{\mathrm{wet}}-w_{\mathrm{dry}}}{D_{\mathrm{iso}}}+w_{\mathrm{dry}}/D_{\mathrm{PTFE}}}$$

where *w*_wet_ is weight of the wet membrane, *w*_dry_ is weight of the dry membrane, *D*_iso_ is isopropanol density and *D*_PTFE_ is density of the polymer.

The tortuosity factor *τ* (^b^) was then deduced from the porosity–tortuosity relationship defined [47] as:

(3) $$\tau =\frac{{\left(2-\varepsilon \right)}^{2}}{\varepsilon }$$

The specific exchange surface area (^c^) was determined from the membrane contactor properties specified by Polymem. 

### 2.5. Ozonation Pilots 

#### 2.5.1. Description of the Pilot Ozonation System: Membrane Contactor with Liquid in Closed Loop

**Figure 2:**
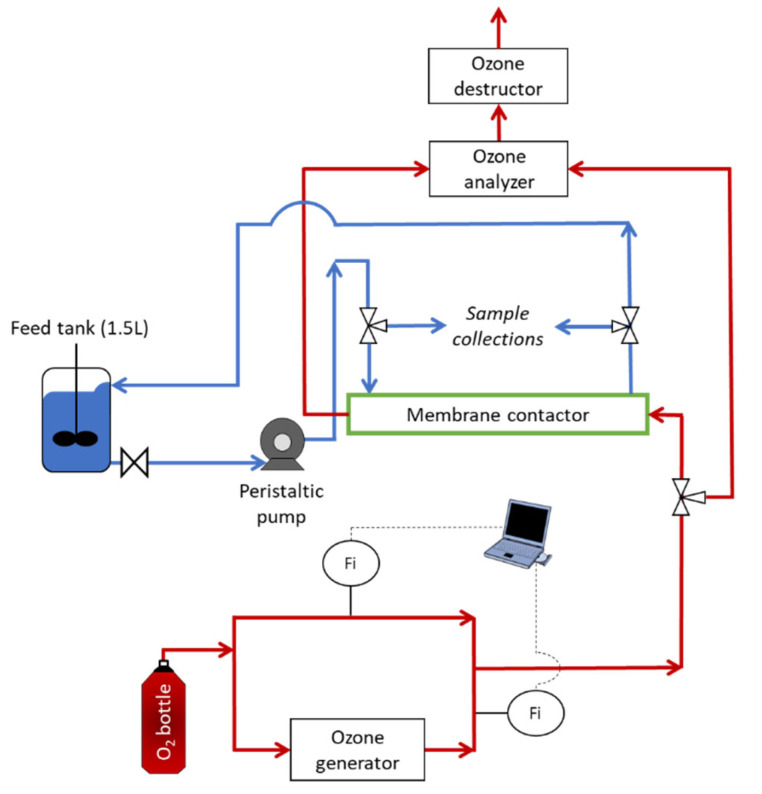
Flowsheet of the ozonation pilot; liquid in closed loop; gas in open circuit (red: gas stream; blue: liquid stream).

The experimental ozonation pilot is described in Figure 2. The liquid flowed into the pilot from a thermostatically-controlled (20 °C) stirred 1.5 L glass tank. Liquid circulation was driven by a peristaltic pump (Watson Marlow 323). Samples were collected one tap upstream and one tap downstream of the membrane contactor. The membrane contactor was continuously fed by an oxygen/ozone mixture. Ozone was produced from a lab-grade pure oxygen tank and an ozone generator (BMT 803 N). Due to electrovalves connected to a computer, ozone was then diluted with the oxygen to achieve the desired gas flowrate and the desired ozone concentration (in the gas phase) before entering in the membrane contactor. An ozone gas analyzer (BMT 964) preceded by dehumidification allowed monitoring ozone gas concentration at the inlet of the membrane contactor. Once the experiment was started, the ozone gas analyzer was used to analyze ozone gas concentration at the outlet of the membrane contactor. Residual ozone was then eliminated by the ozone destructor using active carbon. During the ozonation, ozone was transferred from the liquid phase to the gas phase due to a concentration gradient.

The configuration with liquid in closed loop allowed the increasing of the residence time in the membrane contactor and also served for the study of the bromate formation. Indeed, when the liquid was in open loop, there was no change in the bromide concentration due to the very short residence time (<3 s) in the contactor and the lack of sensitivity of the ion chromatography system. No bromates were detected when the open loop was used. 

This liquid in closed loop configuration was also used during the preliminary step of the experiments with the targeted MPs, which led to an equilibrium between the MPs and the system (i.e., pipework, membrane contactor and feed tank) by leaving the MPs time to adsorb to the system before beginning the ozonation process. During this preliminary step, compressed air was circulating inside the fibers in order to avoid liquid penetration into the membrane pores. The concentrations of the targeted MPs were measured after this preliminary step, and the measurements were considered as the initial concentrations for the ozonation process study. The study of MP removal, therefore, factored out any removal due to potential adsorption on the system (i.e., MPs removal was only due to the ozonation process).

#### 2.5.2. Description of the Pilot Ozonation System: Membrane Contactor with Liquid in Open Loop

The major variation with the pilot described above was that the liquid circulated in open loop, and thus only once in the membrane contactor. At the outlet and in order to prevent the ozone degassing, the liquid was recovered in KI solution. A thermostatically controlled (20 °C) stirred 30 L stainless steel tank was used to achieve the steady-state values. This open-circuit configuration led to simplified mass balances compared to the configuration with the liquid in closed loop, and was thus used to study removal of the targeted MPs. A duplicate was made for each experiment, and a triplicate was made for the reference test (identified in Table 5 by number 1). Before the start of each experiment, the configuration with the liquid in a closed loop was used in order to achieve the balance between the system (i.e., the membrane contactor and piping) and the MPs solution due to potential adsorption of MPs on the system. At the beginning of each experiment, three samples were collected in the tank and another three samples were collected via the tap located at the inlet of the membrane contactor. Once steady state was achieved, six samples of liquid were collected at the outlet of the membrane contactor.

#### 2.5.3. Description of the Pilot Ozonation System with a Bubble Column (Semibatch Reactor)

**Figure 3:**
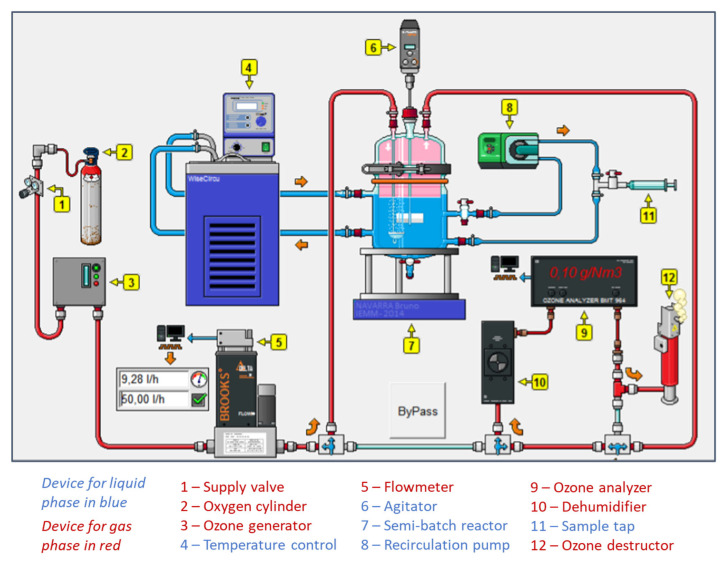
Scheme of the pilot ozonation system with a bubble column.

To compare the performance of the membrane contactor against a conventional process, we also ran experiments using a thermostatically controlled (20 °C) stirred 4 L semibatch bubble reactor with a porous diffuser (Figure 3). Samples were collected using a recirculating pump. The oxygen/ozone mixture was produced from pure oxygen with an ozone generator (BMT 803 N) in the same way as with the pilot ozonation system with the membrane contactor described above. An electrovalve connected to a computer was used to set the gas flowrate. The desired ozone concentration of the gas mixture was obtained by regulating manually the amount of ozone produced with a setting knob on the generator. In the same way as with the pilot ozonation system with the membrane contactor, an ozone analyzer (BMT 964) allowed to monitor the ozone concentration in the gas phase at the inlet or at the outlet of the reactor after dehumidification (to protect the device) due to a by-pass. 

### 2.6. Exposure to Hydroxyl Radicals and Molecular Ozone

Exposure to hydroxyl radicals (M.min) was determined by tracking pCBA, such that:

(4) $$\int_{0}^{t}C_{HO{}^{\circ},liq\;}dt=\frac{1}{k_{OH-pCBA}}\times \ln\left(\frac{C_{p\mathrm{CBA},\mathrm{in}}}{C_{p\mathrm{CBA}}}\right)$$

where $C_{p\mathrm{CBA}}$ is concentration of p-CBA in the water at a given point in time (mol.L^−1^ or g.L^−1^), $C_{p\mathrm{CBA},\mathrm{in}}$ is initial concentration of p-CBA in the water (mol.L^−1^ or g.L^−1^) and $k_{OH-pCBA}$ is the rate constant of p-CBA reaction with HO° (= 5 × 10^9^ M^−1^.s^−1^; [48]). In order to not disturb the water system, and thus not produce misleading results, $C_{p\mathrm{CBA},\mathrm{in}}$ had to be not too high [49].

Exposure to molecular ozone (M.min) was defined by $\int C_{O3,liq\;}dt$, where $C_{O3,liq}$ is the dissolved ozone measured with the indigo method. 

### 2.7. Objectives, Global Parameters and Ozonation Conditions of the Experiments 

#### 2.7.1. Ozonation Experiments for the Removal of Targeted Micropollutants through a PTFE Hollow Fiber Membrane Contactor

Initial concentrations are reported as means and standard deviations (in brackets) with the global parameters for each experiment (Table 5). In the results of these experiments (Section 3.1.), concentrations, removals and fluxes of MPs are reported as means and standard deviations (in brackets in tables and error bars in figures), as well as DOC, COD, SUVA254 index and dissolved ozone concentration. Bromides, bromates and pH are reported with their uncertainties of measurement. Note that standard deviations were calculated from duplicates and, therefore, are not statistically significant. Only experiment number one (i.e., the reference) was triplicated.

**Table 5:** Global parameters and objectives of the experiments with the targeted MPs.

Experiment	1	2	3	4	5	6	7
**Objective**	Reference	Variation in gas concentration	Variation in gas concentration	Variation in liquid flowrate	3 successive passages of the liquid through the membrane contactor (variation in the residence time)		
**Qliq** **(L.h^−1^)**	46.2	46.2	46.2	92.3	47.8		
**Qgas** **(L.h^−1^)**	8						
**C**_**O3,g,inlet**_ **(g.Nm^−3^)**	14.7 (0.1)	22.8 (0.4)	30.6 (0.2)	15.2 (0.5)	14.9 (0.1)		
**pH**	7.9 ± 0.1	7.5 ± 0.1	8.0 ± 0.1	7.5 ± 0.1	7.6 ± 0.1)		
**C_CBZ inlet_ (mg.L^−1^)**	1.92 (0.12)	1.98 (0.02)	2.22 (0.1)	1.92 (0.03)	1.98 (0.03)	1.54 (0.07)	1.31 (0.07)
**C_SUL inlet_ (mg.L^−1^)**	1.95 (0.11)	1.91 (0.02)	2.04 (0.06)	1.85 (0.07)	1.84 (0.03)	1.42 (0.08)	1.16 (0.08)

In the experiments, pH varied from 7.5 to 8 ± 0.1 depending on the pH of the water collected at the outlet of the WWTP.

#### 2.7.2. Ozonation Experiments for the Study of Bromate Production: PTFE Hollow Fiber Membrane Contactor Technology versus Bubble Reactor

Bromates cannot be completely avoided as soon as the concentration of dissolved ozone increases, which is inevitable during the ozonation with a bubble reactor [30]. However, there are potential solutions to minimize bromate formation. In this section, the objective was to evaluate the production of bromates when using the membrane contactor during ozonation of real-treated wastewater that had been spiked with 3 mg.L^−1^ of bromides. This bromide concentration was very high compared to real water and was used here in an effort to overcome the problem posed by the LOD and LOQ of the analytical methods used (ion chromatography). The experiments were thus done in very unfavorable conditions when the objective is to minimize bromate production. 

The ozonation conditions in the membrane contactor were as follows: gas flow-rate of 30 L.h^−1^, liquid flow-rate of 71.5 L.h^−1^ and ozone concentration at the gas-phase inlet of about 10 g.Nm^−3^. The initial volume of liquid flowing in the pilot was 1.5 L, decreasing over time due to the samples. pH of the liquid was measured as 8.2 ± 0.1. A duplicate was run in order to estimate the standard deviation. In the graphs of this section, when no specification is provided, the results are those of the downstream tap.

In order to compare the results obtained here to those of a conventional reactor, a bubble column was also used in semibatch mode (i.e., gas flowing in the form of bubbles from a porous diffusor, while liquid was not flowing). The ozonation conditions in the bubble reactor were as follows: gas flow-rate of 30 L.h^−1^ and ozone concentration at the gas-phase inlet of about 9.2 g.Nm^−3^. The initial volume of liquid flowing in the pilot was 3 L, decreasing over time due to the samples. pH of the liquid was then measured as 8.2. A duplicate was run in order to estimate the standard deviation. Results are reported in the figures of Section 3.2. as means and uncertainties of measurement as error bars. 

## 3. Results and Discussion

### 3.1. Study on Ozonation of Targeted Micropollutants through a PTFE Hollow Fiber Membrane Contactor

#### 3.1.1. Effect of the Ozonation Process on the Global Parameters

**Figure 4:**
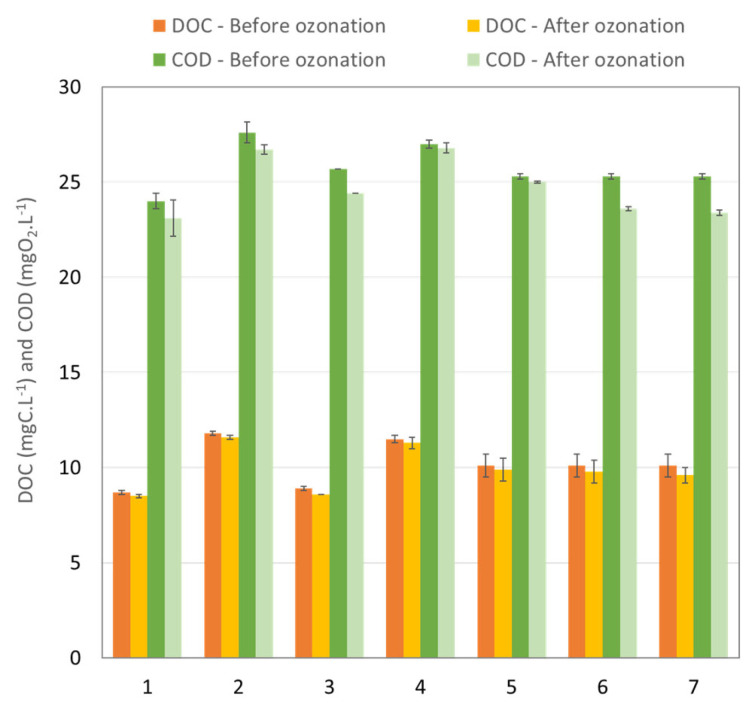
DOC and COD before and after each ozonation experiment. The number in the *x*-axis corresponds to the number of the experiments in Table 5.

**Figure 5:**
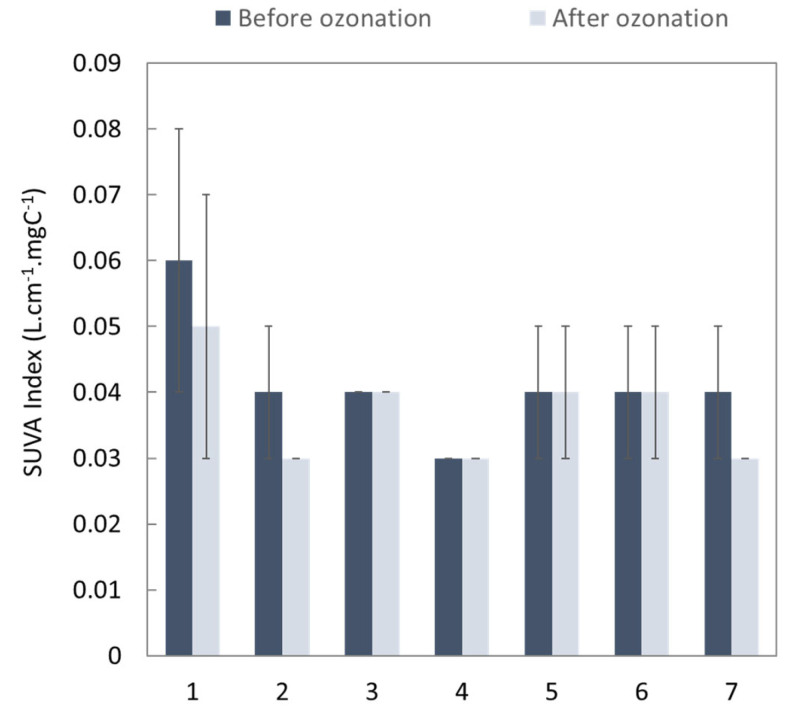
SUVA_254_ index before and after each ozonation experiment. The number in the *x-*axis corresponds to the number of the experiments in Table 5.

During the ozonation process, the ozone does not react only with the MP but also with the carbon or ions found in the matrix under study. Figure 4 and Figure 5 summarize the global parameters of the water before and after each experiment (described in the Table 5), i.e., specifically the dissolved organic carbon (DOC), chemical oxygen demand (COD) and specific ultraviolet absorbance at 254 nm (SUVA_254_ index). 

The differences between DOC before ozonation for each experiment (Figure 4) are explained by the composition of the water collected at the outlet of the WWTP, which depends on the season. The ozonation process led to very little reduction in DOC. This can be explained by the short residence time in the contactor, which did not enable complete oxidation of all compounds. In particular, the MPs were transformed into metabolites, which are also organic compounds. For this same reason, the aromaticity of the water, as characterized by the SUVA_254_ index (Figure 5), which is representative of the quantity of double bonds and is typically impacted during conventional ozonation processes, did not change during the experiments or only changed within the standard deviation. Only COD (Figure 4) showed a significant (but low) decrease, reducing by up to 7.5% in the case of recirculation after the third run (eighth experiment). These findings highlight the importance of residence time as a factor for improving the overall quality of the treated water, as a duration of 3s is too short to have an impact on the global parameters but enough to remove a substantial amount of MPs. 

#### 3.1.2. Effect of Ozone Concentration

In this series of experiments, the concentration of ozone in the gas phase at the inlet of the contactor was varied (i.e., set at 15 g.Nm^−3^, 22.5 g.Nm^−3^ and 30 g.Nm^−3^ in columns one, two and three, respectively, in Table 5). Liquid flow-rate was fixed at 46.2 L.h^−1^ and the gas flowrate at 8L.h^−1^. The concentration of CBZ and SUL at the inlet was 1.92 ± 0.12 and 1.95 ± 0.11, respectively.

**Figure 6:**
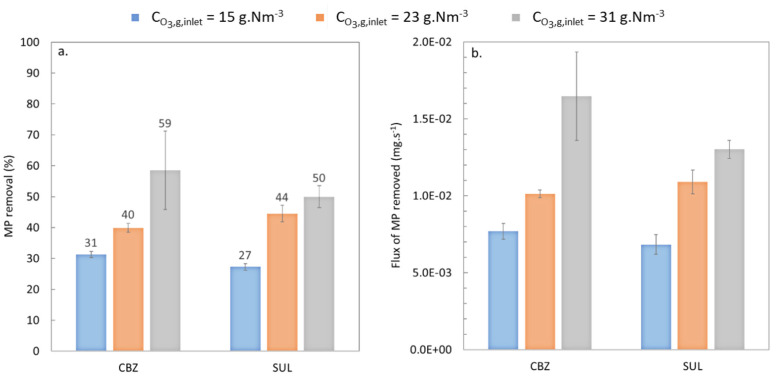
Abatement of CBZ and SUL at ozone concentrations in the gas-phase inlet of 15, 23, and 31 g.Nm^−3^, a liquid flowrate of 46.2 L.h^−1^ and a gas flowrate of 8 L.h^−1^ at neutral pH.

Figure 6a shows the abatement of each targeted MP based on Equation (1) for the different concentrations of ozone in the gas phase. Increasing ozone concentrations led to increasing rates of removal for all the MPs studied. This confirms results obtained in previous work using the same membrane contactor, where this parameter was highlighted as having a significant impact on ozone transfer [39].

Figure 6 clearly shows that SUL abatement was similar to CBZ abatement (within the standard deviations) for the tested conditions (ozone concentrations in the gas phase inlet of 15, 23, and 31 g.Nm^−3^, liquid flowrate of 46.2 L.h^−1^ and gas flowrate of 8 L.h^−1^). Indeed, molecular mechanisms are promoted when k_O3_ > 10^4^ M^−1^.s^−1^ and the kinetic rate constants of the CBZ reaction with ozone and the SUL reaction with ozone are 3.0 × 10^5^ M^−1^.s^−1^ and 4.2 × 10^5^ M^−1^.s^−1^, respectively [19,36]. Therefore, the molecular mechanism was the main route of action for removal of the targeted MPs, and the reaction of CBZ with ozone was as fast as the reaction of SUL with ozone. 

The abatement percentages were lower than those achievable with conventional ozonation processes (100% for CBZ and 97–99% for SUL [41]). Note, however, that the removal rates achieved here were for a very short residence time of 2.8 s in the membrane contactor instead of between 10 and 30 min in a traditional process (at full WWTP scale) [50]. Part of the study by Mathon et al. investigating the ozonation of MPs used batch experiments with real-treated wastewater [41], where A volume of 30 L of water was spiked with 0.01 mg.L^−1^ of CBZ and A specific dose of ozone of 1.6 gO_3_.gDOC^−1^ was transferred during 15 min. Mathon et al. showed that 100% of CBZ was removed in about 125 s, corresponding to an MP removal flux of 2.4 × 10^−3^ mg.s^−1^, which is lower than that found here. Furthermore, a CBZ abatement of about 41% was achieved in 60 s, which is more than 20 times longer than here study for a similar abatement percentage. Note too that the specific dose of ozone transferred in the experiments, reported in Figure 6, was comprised between 0.14 and 0.21 gO_3_.gDOC^−1^ instead of 1.6 gO_3_.gDOC^−1^ in Mathon et al., which could further explain the lower MP abatement rates achieved here.

In addition, despite the relatively low percentage rates of MP abatement, the quantity of MPs actually removed was high, as our experiments started with higher initial concentrations than in reality (i.e., at the outlet of the WWTP) or in other studies where MP load concentrations vary from ng.L^−1^ up to µg.L^−1^ scale [34,35].

Moreover, the results reported here were obtained with a membrane contactor having an exchange surface area of just 0.107 m². One of the big advantages of membrane contactors is their design flexibility, as the surface area of the membrane (i.e., the exchange surface) can easily be increased while keeping a very low footprint compared to conventional processes. Both the parameters of the process and the design of the membrane contactor must be optimized in order to better compare the removal rates obtained with this process and those obtained with conventional processes.

#### 3.1.3. Effect of the Liquid Flowrate

**Figure 7:**
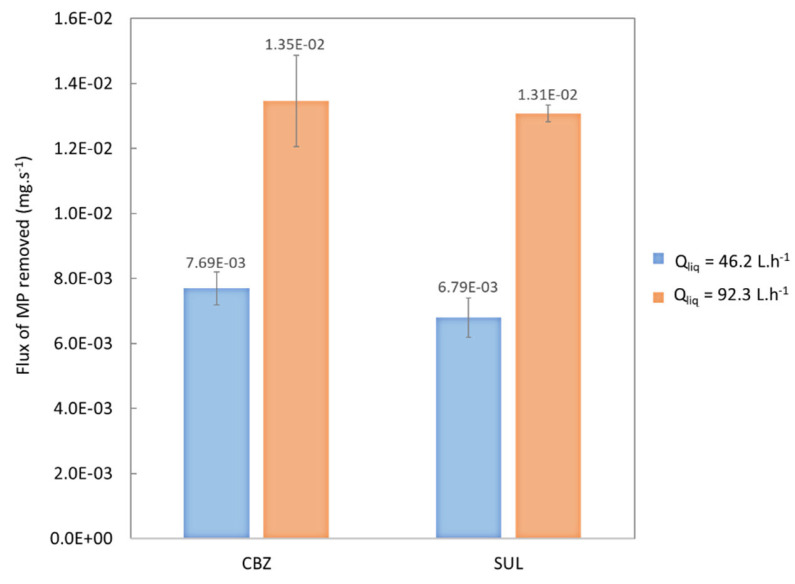
Flux of CBZ and SUL removed at a liquid flow-rate of 46.2 L.h^−1^ and 92.3 L.h^−1^, an ozone concentration at the gas-phase inlet of 15 g.Nm^−3^ and a gas flow-rate of 8 L.h^−1^ at neutral pH.

In order to study the effect of liquid flowrate, experiments were realized at two liquid flowrates of 46.2 L.h^−1^ and 92.3 L.h^−1^, a gas flowrate of 8 L.h^−1^ and an ozone concentration at the gas phase inlet of about 15 g.Nm^−3^. The results are reported in Table 6 and Figure 7 as means and standard deviations (in brackets).

Figure 7 shows that more MPs were removed when the liquid flow-rate was doubled. This is explained by that fact that when the liquid flowrate is increased, there is a higher flux of transferred ozone due to a decrease in transfer resistance in the boundary layer between the liquid and the membrane. Indeed, this boundary layer thickness decreases with increasing flow-rate of the liquid. MPs removal was thus better at a higher liquid flow-rate despite the shorter residence time in the membrane contactor.

**Table 6:** MP elimination rates and transferred ozone in experiments run at the two liquid flow-rates.

Liquid Flowrate	46.2 L.h^−1^		92.3 L.h^−1^	
**Transferred ozone** **(mg.min^−1^)**	0.92		1.80	
**MP**	CBZ	SUL	CBZ	SUL
**Concentration of MPs at the inlet** **(mg.L^−1^)**	1.92 (0.12)	1.95 (0.11)	1.92 (0.04)	1.85 (0.07)
**Concentration of MPs at the outlet** **(mg.L^−1^)**	1.32 (0.09)	1.42 (0.06)	1.39 (0.03)	1.34 (0.08)
**Flux of MPs removed** **(mg.s^−1^)**	7.69 × 10^−3^ (5.13 × 10^−4^)	6.79 × 10^−3^ (5.98 × 10^−4^)	1.35 × 10^−2^ (1.41 × 10^−3^)	1.315 × 10^−2^ (2.56 × 10^−4^)

Figure 7 shows that the flux of SUL removed was similar to the flux of CBZ removed (within the standard deviations). In the same way shown in Figure 6, these results are consistent with the assumption that the molecular mechanism was the main way of action for the MP removal.

#### 3.1.4. Effect of Residence Time: Recirculation System

The effect of the recirculation of the water was also studied by increasing residence time in the membrane contactor from 2.7 s at the first run, to 5.4 s at the second run and then 8.1 s at the third run. These experiments were different from the protocol followed in Section 3.1.2. During these experiments, the liquid flow-rate was fixed at 47.8 L.h^−1^, gas flowrate was fixed at 8 L.h^−1^ and ozone concentration at the gas-phase inlet was about 15 g.Nm^−3^. These experiments were equivalent to a scheme with three membrane contactors configured in series. The objective was to simulate a hypothetical industrial installation, which seemed realistic even though not optimized in terms of design, process parameters and number of cycles. Note that the increase in liquid flow-rate had an impact on residence time (decreased); however, it mostly impacted the liquid flow which increased the ozone transfer despite a shorter residence time (Table 6). 

**Figure 8:**
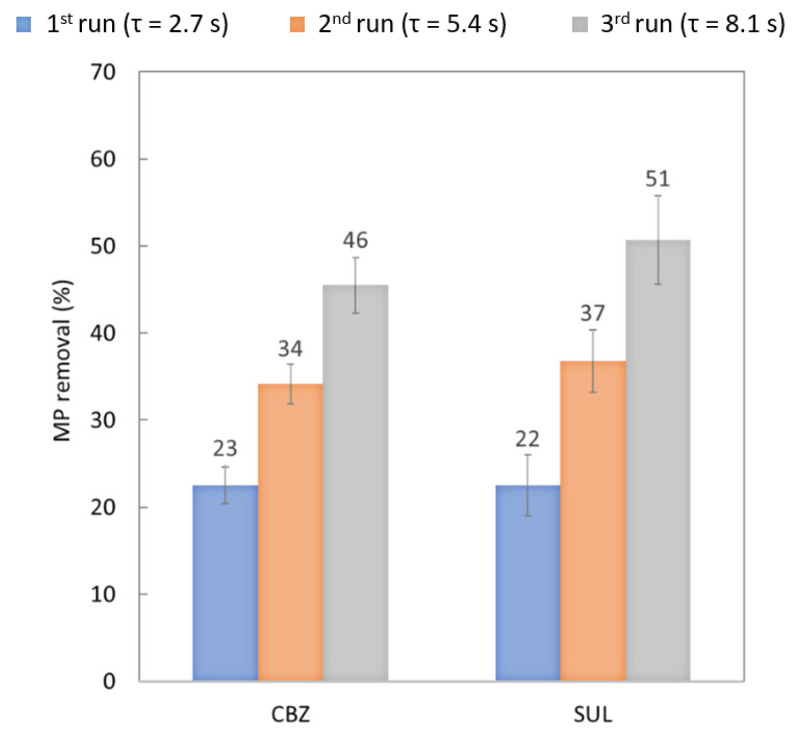
Percent removal of MPs calculated from the inlet concentration before the 1st run of experiments with recirculating water with an ozone concentration at the gas-phase inlet of 15 g.Nm^−3^, a liquid flow-rate of 47.8 L.h^−1^ and a gas flow-rate of 8 L.h^−1^ at neutral pH.

Figure 8 charts the abatement of the targeted MPs after each water run in the contactor. The calculation was based on the initial MP concentration at the inlet of the contactor before the first run. Figure 8 clearly highlights that percent removal was better after each recirculation for all the MPs studied. The first run achieved roughly 23% abatement of the targeted MPs. In the second and third runs, SUL abatement was non-significantly higher than CBZ abatement (i.e., in the standard deviations). After three runs, the system achieved 46% abatement of CBZ and 51% abatement of SUL. A higher residence time, therefore, has a positive impact on MP removal. However, the evolution of the abatement was not proportional to the evolution of the residence time as the decreasing MP concentrations led to slower reaction rate kinetics. For instance, CBZ removal doubled when residence time was tripled. This highlights the necessity to optimize the residence time in a membrane contactor in order to find the best trade-off between treatment duration and MP concentration at the outlet.

**Figure 9:**
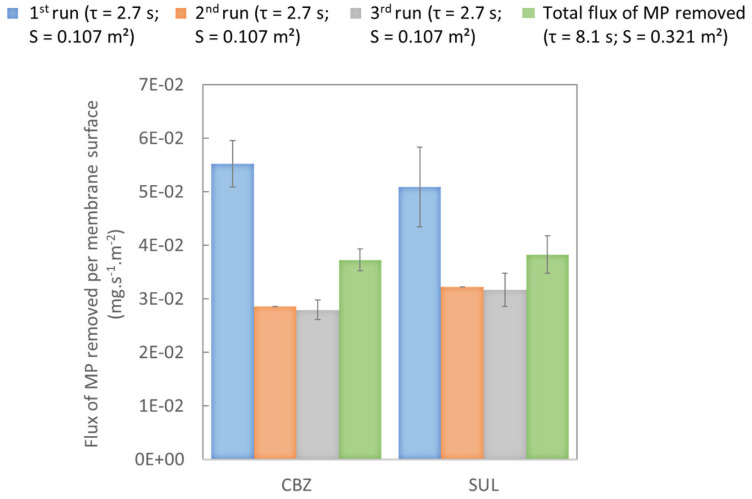
Flux of MPs removed per membrane surface during each run and total flux removed after 3 runs (mg.s^−1^.m^−2^) in experiments with recirculating water with an ozone concentration at the gas-phase inlet of 15 g.Nm^−3^, a liquid flow-rate of 47.8 L.h^−1^ and a gas flow-rate of 8 L.h^−1^ at neutral pH.

Figure 9 plots the flux of MP removed per membrane surface during each run in the steady state (in mg.s^−1^.m^−2^). Unlike the results presented in Figure 8, the calculation was based on the inlet and outlet of each run. Note that the fluxes of MP removed were significantly higher during the first run compared to the second and third runs, where the fluxes were similar (i.e., within the standard deviations). However, the flux of ozone transferred, which was calculated from a mass balance on the gas-phase, was approximately the same at each run, decreasing only slightly: 0.80 (± 0.05) mgO_3_.min^−1^ at the first run, 0.79 ± 0.07 mg O_3_.min^−1^ in the second run and 0.74 ± 0.06 mg O_3_.min^−1^ in the last run. At the same time, the concentration of residual ozone was about the same for each run (Table 7). The difference between the first run and the following runs is, therefore, explained by slower reaction-rate kinetics due to a lower concentration of MP. 

Figure 9 also plots total flux of MPs removed after three runs per membrane surface (in mg.s^−1^.m^−2^), which was calculated from concentration at the inlet of the first run and was similar for the two MPs. 

CBZ and SUL flux removals were similar in each run. As CBZ is almost as reactive with molecular ozone as SUL (3.0 × 10^5^ M^−1^.s^−1^ and 4.2 × 10^5^ M^−1^.s^−1^, respectively), as shown before, these results further support the molecular mechanism as the main mode of removal of the targeted MPs during ozonation. In the case of MPs that are less reactive to ozone, both molecular and radical mechanisms could occur simultaneously (for 10^2^ < k_O3_ < 10^4^) or radical mechanisms may be predominant (for k_O3_ < 10^2^) [19,36].

#### 3.1.5. Residual Ozone and Bromate Formation during Removal of Selected MPs

Table 7 reports the bromide concentrations before and after each ozonation experiment and the concentrations of bromate and residual ozone (i.e., dissolved ozone) after ozonation. The ozonation conditions applied for each experiment are described in Table 5. Note that no bromate was detectable after ozonation. This is explained by the combination of a very low residual ozone concentration and a very short residence time, both of which are parameters that bromate formation (see below). The LOD of the bromates was high (0.1 mg.L^−1^), which makes it instructive to analyze the evolution of bromide concentration (for which relative standard deviation was 2%). Indeed, the graph plotted in Figure S1 shows that one mole of bromate was formed for one mole of bromide consumed. Therefore, a decrease of 2% of the initial bromide concentration led to the formation of between 0.01 and 0.05 mg.L^−1^ of bromates, depending on the initial bromide concentration (between 0.3 and 1.6 mg.L^−1^), that was not directly detectable from bromates’ analyses due to their high LOD. No reduction of bromides was found in any of the experiments, confirming that no (or very little) bromate was formed even in the experiments involving several runs through the membrane contactor (i.e., involving longer residence times).

**Table 7:** Concentrations of bromides, bromates and residual ozone before and after the different experiments.

Experiment	1	2	3	4	5	6	7
**C_Br-_** **before ozonation (mg.L^−1^)**	0.30 ± 0.01	1.10 ± 0.02	0.28 ± 0.01	0.30 ± 0.01	1.56 ± 0.03		
**C_Br-_** **after ozonation** **(mg.L^−1^)**	0.31 ± 0.01	1.10 ± 0.03	0.28 ± 0.01	0.30 ± 0.01	1.55 ± 0.03	1.55 ± 0.03	1.55 ± 0.03
**C_BrO3-_** **after ozonation** **(mg.L^−1^)**	< LOD	< LOD	< LOD	< LOD	< LOD	< LOD	< LOD
**C_O3,liq_** **(mg.L^−1^)**	0.05 (0.01)	0.08 (0)	0.08 (0.01)	0.07 (0)	0.04 (0.03)	0.06 (0.03)	0.05 (0.03)

### 3.2. Bromate Minimization: Membrane Contactor Technology versus Bubble Reactor

#### 3.2.1. Formation of Bromates

The stoichiometric ratio between the bromates produced and the bromides consumed is plotted in Figure S1. The graph shows that one mole of bromate was formed for one mole of bromide consumed, irrespective of the process used (conventional bubble reactor ozonation or experimental membrane contactor process). The substantial difference between the results obtained according to process used is explained by the initial concentration of bromide.

The matrix studied, which came from the outlet of the WWTP at La Grande-Motte and contained about 0.3 mg.L^−1^ of bromide (= 3.75 mol.L^−1^) during winter (and even more during summer), could therefore reach a concentration of 3.75 mol.L^−1^ of bromate (=4.8 mg.L^−1^) after ozonation. This concentration is significantly higher than that authorized in drinking water (maximum of 10 µg.L^−1^), but, as yet, there are no defined regulations governing bromate levels in wastewater.

**Figure 10:**
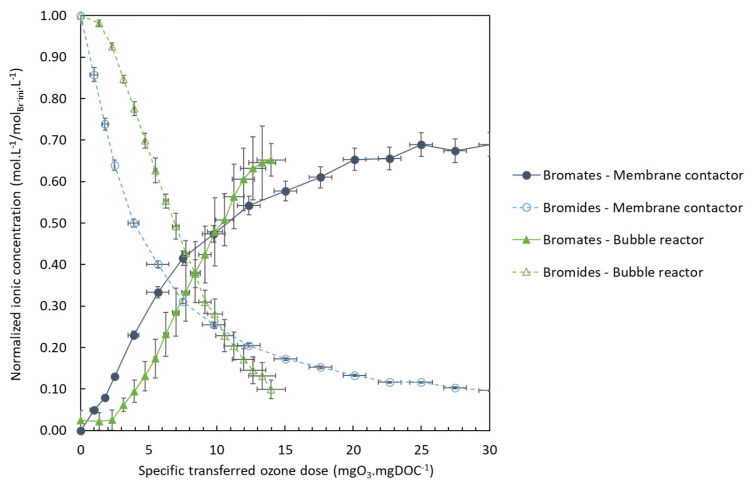
Normalized concentrations of bromate and bromide at pH 8.2 as a function of specific dose of ozone transferred (mgO_3_.mgDOC^−1^).

Figure 10 plots the evolution of the bromide and bromate normalized concentrations as a function of specific dose of ozone transferred (mgO_3_.mgDOC^−1^), calculated from mass balance analysis on the gas-phase. It provides a comparison between the two processes used and is, therefore, useful to evaluate the value of the membrane contactor as a solution minimize bromate formation. The parameters used were the same for the two processes, including pH, which has a significant impact on the reaction mechanism involving the transformation of bromides into bromates. Only the initial concentration of bromide differed, for the reason stated above, which places the bubble-reactor experiments in disadvantageous conditions for bromate minimization.

Figure 10 depicts two different steps. First, for a specific dose of ozone transferred up to 9.9 mgO_3_.mgDOC^−1^, more bromates were produced using the membrane contactor despite the lower quantity of bromides. Once this dose was exceeded, less bromates were formed with the membrane contactor than with the conventional process. Moreover, during this second step, there was a sharp slowdown in the bromate formation, which tended to stabilize while bromides were still available. Therefore, the use of a membrane contactor to minimize bromate formation is only worthwhile under certain conditions, i.e., a high specific dose of ozone transferred. However, the membrane contactor only achieved this dose after a long experimental time (i.e., approximately 3 hours), and thus a lot of recirculation of the water in the contactor and a high residence time. In practice, when the pilot-system configuration is an open loop, residence time in the membrane contactor would only be a few seconds, corresponding to a very low dose of ozone transferred. It would thus appear that the conventional reactor (i.e., the bubble reactor) is a more appropriate technology than the membrane contactor for minimizing bromate formation during a long ozonation process. Nevertheless, the very short contact time (i.e., just seconds) offered by the membrane contactor, which leads to good MP abatement performances (see Section 3.1), could potentially lead to little or no bromate formation as suggested in the results of Section 3.1.5. (although the LOD was high). Indeed, a large part of the reactions involved in bromate formation are slower than the reactions involved in MP abatement. The mechanism of bromate formation is a very complex with a lot of reactions occurring simultaneously between the compounds of the matrix, the molecular ozone and the hydroxyl radicals [51]. The O_3_-driven transformation of Br^−^ into BrO^−^, which initiates this mechanism, is about 10^2^ M^−1^.s^−1^, which is slower than the reaction rates between O_3_ and the targeted MPs [52]. 

#### 3.2.2. Production of Hydroxyl Radicals

**Figure 11:**
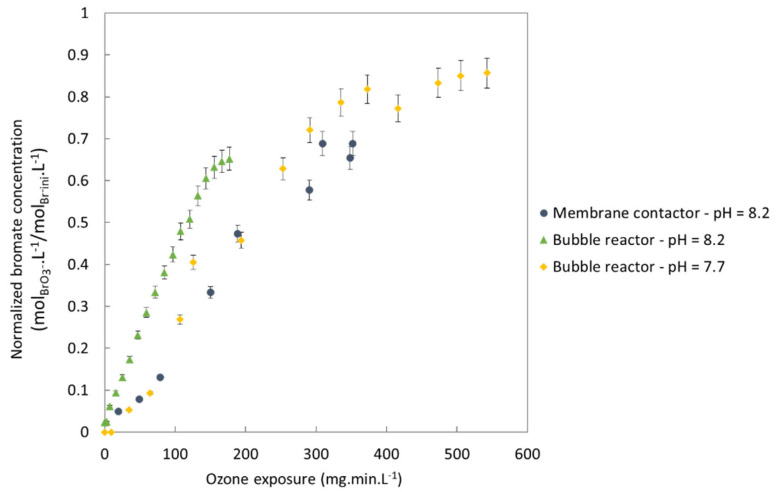
Normalized bromate concentration as a function of ozone exposure during ozonation with a membrane contactor at pH 8.2, a bubble reactor at pH 8.2 and a bubble reactor at pH 7.7.

The representation of bromate and bromide as a function of the dose of ozone transferred fails to account for the decomposition of the dissolved ozone into hydroxyl radicals. Hydroxyl radicals provide strong oxidizing power, which is useful to efficiently remove organic pollutants, and they also come into play (although to a lesser extent than the dissolved ozone) in the mechanism of bromate formation. It is thus important to analyze bromate formation according to ozone exposure, which is known to be a crucial parameter for bromate formation [33]. Figure 11 confirms this and shows that bromate formation is strongly dependent on ozone exposure.

At identical ozone exposure, the semibatch reactor produced more bromates than the membrane contactor. One assumption that might explain this result is the local concentration of the dissolved ozone in the reactors. The bubble reactor is considered as perfectly stirred, leading to a fast homogeneous distribution of the ozone. In the membrane contactor, the dissolved ozone is very concentrated close to the fibers but varies significantly according to location, even reaching zero in some areas (close to the shell wall). This concentration is then diluted and homogenized at the outlet. A future study is planned to more deeply explore the local distribution of the dissolved ozone in a membrane contactor.

**Figure 12:**
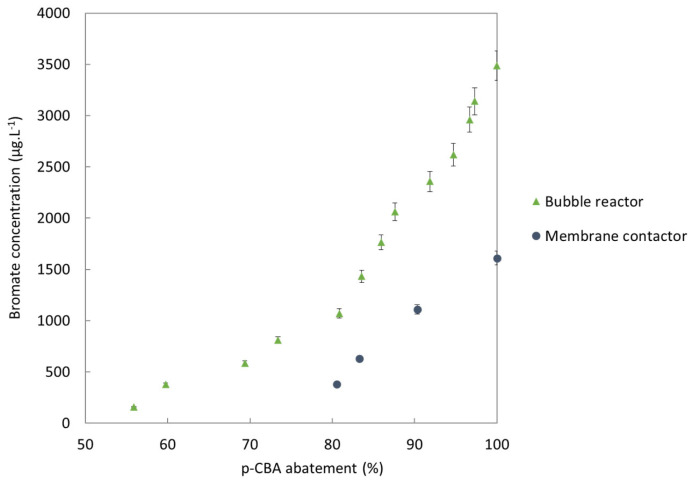
Evolution of bromate concentration as a function of p-CBA abatement according to ozonation process used at pH 8.2.

Figure 11 also illustrates the important impact of pH on bromate formation: more bromates are produced at higher pH values. For a pH at 7.7 instead of 8.2, there was approximately 44% less bromate formed on average, depending on ozone exposure. This coincides with the results reported by Pinkernell and Von Gunten, who found that the decrease of pH from 8 to 6 led to 60% less bromates with a river water at an ozone exposure of 10 mg.L^−1^.min^−1^ [33]. Another study by Chao concluded that pH was the parameter with the highest impact the bromate formation, ahead of ozone exposure, DOC, bromides or ammonium [53]. Control of pH is, therefore, a good solution to minimize bromate formation but hard to put in place in a real-world WWTP.

p-CBA had been added to the matrix in order to track the quantity of hydroxyl radicals produced during the ozonation process. Figure 12 shows that for a same initial p-CBA concentration, when complete elimination of the p-CBA (and thus the same production of hydroxyl radicals) was achieved, the membrane contactor produced 1.6 mg.L^−1^ of bromates, whereas the standard bubble reactor produced 3.5 mg.L^−1^. Moreover, the complete elimination of p-CBA was achieved for a specific dose of ozone transferred of 5.7 mgO_3_.mgDOC^−1^ with the membrane contactor vs. 11.3 mgO_3_.mgDOC^−1^ with the bubble reactor. The membrane contactor thus required a lower specific dose of ozone transferred to produce the same amount of hydroxyl radicals. This result points to the possibility of using the membrane contactor to produce a significant quantity of HO° in order to efficiently remove MPs while minimizing bromate formation compared to a conventional reactor during the ozonation of WWTP effluents. 

The curves plotted in Figure 12 share a similar profile to the results found by Merle et al., who also studied ozonation via a membrane contactor [34]. We cannot reliably compare our values against those found by Merle et al. as the initial concentrations of bromide were very different and Merle et al. also used peroxone (i.e., not only ozone). However, their study did demonstrate the value of decreasing the ozone concentration in the gas phase to minimize bromate production. They also proved that a longer residence time in the contactor (i.e., a lower liquid flow-rate) produced more bromates but also more hydroxyl radicals. Roustan had already highlighted the importance of residence time in the contactor as a factor in bromate formation [54]. In that sense, the membrane contactor presented in this work seems to make an ideal configuration due to its very short liquid residence time (<2 s for a liquid flow-rate of 71.5 L.h^−1^ in a single pass). Furthermore, no bromate was detected after the experiments with the liquid in an open-loop configuration. However, the LOD of the ion chromatography protocol used was about 0.1 mg.L^−1^, which is higher than the regulatory limit of 10 µg.L^−1^ in drinking water.

Although HO° production is very important for a good removal of organic pollutants, HO° also contributes to bromate formation. Indeed, bromates are produced by both molecular and radical pathways. The ratio between each mechanism depends on the quality of the water and the parameters of the ozonation process. Von Gunten et al. showed that the radical mechanism predominates the molecular mechanism in the case of short ozone exposure and low bromide concentration [55]. 

## 4. Conclusions

Using the membrane contactor to treat carbamazepine and sulfamethoxazole in real wastewater with very short contact times can, under certain conditions, achieve substantial removal yields depending on the reactivity of the micropollutant to ozone and hydroxyl radicals. At the same time, as the residence time is extremely short, the process produces little or no bromate. 

For a residence time of less than 3s, the experimental membrane contractor process achieved up to 59% carbamazepine abatement and 50% sulfamethoxazole abatement depending on the conditions tested. When contact time was lengthened to about 8s (but with a lower ozone concentration in the gas phase than for the previous result with a residence time of less than 3s), percent removal reached 46% and 51%, respectively, without optimization of the process parameters. Better abatement yields would almost certainly be achieved by using a higher ozone concentration in the gas-phase and a higher liquid flow-rate but also lower initial MP concentrations (the MP concentrations used in this work were not representative of real concentrations at the outlet of a WWTP due to the LOD and LOQ of the material used for the analyses).

In addition, the very short contact time in the membrane contactor is likely to positively prevent bromate formation, as the ozonation reactions with MPs are faster than the reactions involved in the mechanism of bromate formation. 

Nevertheless, it is difficult to firmly conclude that less bromates are produced with a membrane contactor than with a bubble reactor under the experimental conditions used in this work. For a low dose of transferred ozone, the bubble reactor emerged as the better solution to minimize bromate formation, despite experimental conditions promoting the production of bromates, such as higher initial bromide concentration and higher DOC than in the experiments with the membrane contactor. Moreover, irrespective of the reactor used, the initial conditions of the experiments were very unfavorable to bromate minimization, with a very high initial bromide concentration compared to other studies and to concentrations found in real wastewater. 

This study highlights that a membrane contactor process produces less bromate than a semibatch bubble reactor for the same production of hydroxyl radicals and the same ozone exposure. Membrane contactors, therefore, emerge as a good alternative to bubble columns to simultaneously treat refractive pollutants in wastewater and minimize bromate formation.

## Figures and Tables

**Table 1:** Main characteristics of the targeted micropollutants (^a^ [40], ^b^ [41], ^c^ [42]).

Compound	Formula	MW (g.mol^−1^)	k_O3_ (M^−1^.s^−1^)	k_OH_ (M^−1^.s^−1^)	Log K_ow_	Solubility in Water (25 °C, mg.L^−1^)	Semi-Developed Formula
Carbamazepine (CBZ)	C_15_H_12_N_2_O	236	3.0 × 10^5 a^	8.8 × 10^9 b^	2.45 ^b^	18 ^b^	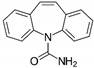
Sulfamethoxazole (SUL)	C_10_H_11_N_3_O_3_S	253	4.2 × 10^5 c^	3.2 × 10^9 b^	0.89 ^b^	610 ^b^	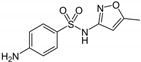

Potassium indigotrisulfonate (C_16_H_7_K_3_N_2_O_11_S_3_, MW = 617 g.mol^−1^) and sodium sulfite (Na_2_SO_3_, MW = 126 g.mol^−^) were used as ozone-scavenging reagents. Both were stored at 4 °C. Sodium bromide, anhydrous (BrNa, MW = 103 g.mol^−1^) and 4-chlorobenzoic acid (p-CBA) (C_7_H_5_ClO_2_, MW = 157 g.mol^−1^) were used to spike the matrix during the experiments on bromate formation. Both were stocked at room temperature.

**Table 2:** Global parameters of the WWTP effluent.

Parameters	Mean Value	Standard Deviation
TOC (mgC.L^−1^)	6.8	0.7
COD (mgO_2_.L^−1^)	18.0	1.0
pH	7.7	0.2
SUVA_254_ (L.cm^−1^.mgC^−1^)	0.02	0.01

**Table 3:** Ionic composition of the WWTP effluent.

Average Ionic Composition (mg/L)			
**Sodium** **(Na^+^)**	88.1 ± 0.8	**Bromate** **(BrO_3_^−^)**	< LOD
**Ammonium** **(NH_4_^+^)**	< LOD	**Chloride** **(Cl^−^)**	155 ± 6
**Potassium** **(K^+^)**	22.6 ± 0.3	**Nitrite** **(NO_2_^−^)**	0.2 ± 0.2
**Magnesium** **(Mg^+^)**	10.1 ± 0.3	**Chlorate** **(ClO_3_^−^)**	0.5 ± 0.3
**Calcium** **(Ca^2+^)**	92.0 ± 0.7	**Bromide** **(Br^−^)**	0.29 ± 0.03
**Sulphate** **(SO_4_^2−^)**	84 ± 10	**Phosphate** **(PO_4_^3−^)**	0.3 ± 0.3

**Table 4:** Technical specifications of the membrane contactor.

PTFE Fibers			
Number *	**65**	Effective Contact Length (cm) *	**60**
Inner/outer diameter (mm) *	0.45/0.87	Effective contact surface (m²)	0.107
Specific exchange surface *a* (m²/m^3^) ^c^	2948	N_2_ permeance (GPU) *	33,904
Porosity ^a^	0.58	Tortuosity ^b^	3.47
**Stainless steel shell**			
Inside diameter (mm) *	9.5	Filling rate *	54.5%

* Values specified by the manufacturer (Polymem, France).

## Data Availability

Not applicable.

## Supplementary Materials

The following supporting information can be downloaded at: https://www.mdpi.com/article/10.3390/membranes13020171/s1, Figure S1: Evolution of the bromate concentration as a function of bromide concentration at pH 8.2; Table S1: Gradient elution timetable used for LC-MS/MS analyses.
