# Supplementary material for: Ozone Diffusion through a Hollow Fiber Membrane Contactor for Pharmaceuticals Removal and Bromate Minimization
Source: Membranes (Basel). 2023 Jan 31;13(2):171. doi: 10.3390/membranes13020171 (PMC9959604; doi:10.3390/membranes13020171)
Supplement: Supplementary file 1 [file membranes-13-00171-s001.zip › membranes-2120527-supplementary.pdf]

Table S1. Gradient elution timetable used for LC-MS/MS analyses

Time (min)	A (%)	B (%)
0.00	100.0	0.0
0.50	100.0	0.0
4.50	0.0	100.0
5.50	0.0	100.0
6.00	100.0	0.0
10.00	100.0	0.0

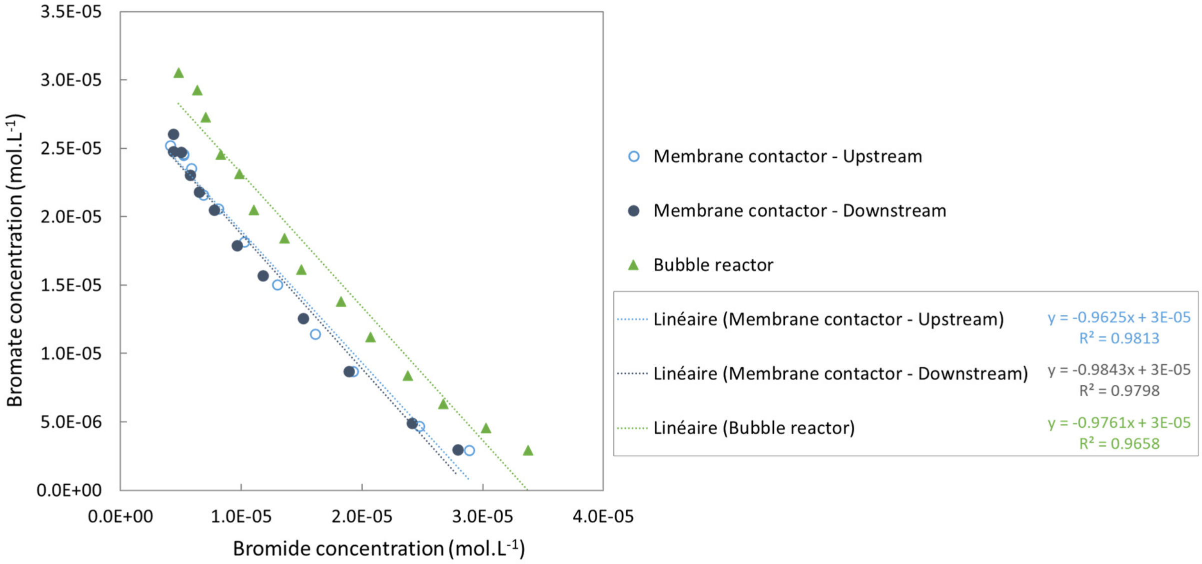

Figure S1. Evolution of the bromate concentration as a function of bromide concentration at pH 8.2.
